# What Can Health Services Researchers Offer Health Systems? Developing Meaningful Partnerships Between Academics and Health System Workers Comment on "Experience of Health Leadership in Partnering with University-Based Researchers in Canada - A Call to ‘Re-imagine’ Research"

**DOI:** 10.15171/ijhpm.2020.07

**Published:** 2020-01-22

**Authors:** Kate Churruca, Louise A. Ellis, Janet C. Long, Jeffrey Braithwaite

**Affiliations:** Australian Institute of Health Innovation, Macquarie University, Sydney, NSW, Australia.

**Keywords:** Knowledge Translation, Embedded Research, Research Partnerships, Health Systems, Australia

## Abstract

As healthcare researchers, we know very well our own experiences on the challenges of partnering with those in the health system to do collaborative, internationally-regarded studies aiming for impact. Bowen and colleagues’ study in Canada empirically examines these issues from the other side, interviewing health system leaders about their perspectives of us researchers, research collaborations and the challenges and opportunities these pose. Based on their findings, they propose a need to re-imagine the contours of research. Inspired by that, in this commentary we examine the context for research partnerships and consider some of the emerging models for fostering more meaningful collaborations between researchers and those working in healthcare systems and organisations. Based on principles of embedded research and researchers, these models—including translational research networks (TRNs) and researcher-in-residence models—rely on a complex interplay of personal and interpersonal factors to be successful.

This commentary is inspired by a recent empirical study of the experiences of health system leaders in partnering with university-based researchers by Bowen, Botting, Graham, MacLeod, de Moissac, Harlos, Leduc, Ulrich, and Knox.^1^ We draw upon their findings, and our own experience and research interests to describe emerging models of research partnership that may facilitate more sustained and meaningful research collaborations between these groups. These forms of partnership involve embedding research and researchers within the healthcare system.

As researchers of health systems, we have sometimes experienced the fraught feeling that comes with having to follow up with a health system research partner after one or a number of emails we sent requesting a meeting or feedback, for example, have gone unanswered and unacknowledged. In emailing again or calling, we walk the tenuous tight-rope of desperately needing their input—or at least their site approval to proceed with a study—before being able to progress our research, while simultaneously wanting to appear collaborative, in control, professional, and authoritative. It is easy to assume—and we often have—that this poor response is down to the peculiarities of our partners, typically senior clinicians or managers, who are at best too time poor to give our research the attention it deserves, and at worst disinterested, with less appreciation than we would hope for the value of the scientific endeavour. The publication of Bowen et al^1^ article on “Experience of Health Leadership in Partnering With University-Based Researchers in Canada: A Call to ‘Re-imagine’ Research” forces us to acknowledge that for every substantive meaningful relationship we have had with those “out in the system,” we researchers have also sometimes forged “partnerships” that are of the more tokenistic, “collaborations on paper” (p. 6) variety, for the purposes of getting our work done.

Bowen et al^1^ look at the other perspective on these issues, using interviews to study the experiences of those health systems leaders who researchers partner with to access research sites and enact change in health systems. It is all too easy to judge these partners as disinterested or giving a low priority to research when they fail to respond, or meet our needs or expectations. Their participants acknowledged the challenges even in promoting the use of research findings. However, we have, for the most part, moved on from simply scratching our heads bewilderingly at *why* clinicians do not engage or use research, to recognising the complexity of the evidence-into-practice journey.^2^

One response has been to recognise that research is far more likely to find purchase in the real world when pulled by end-users, rather than pushed by researchers.^3^ This requires consequential, often long-term partnerships between university-based researchers and those end users, including policy-makers, clinicians, patients and health leaders. At the heart of what makes “A Call to ‘Re-imagine’ Research” important is its explicit empirical exploration of end users’ perspectives, which begins to build the knowledge base for *how* we can make research partnerships worthwhile and meaningful for stakeholders, and ensure they lead to improvements in services and outcomes for patients. Reflecting on some of the issues Bowen and colleagues’^1^ paper raises for the Australian health system, we consider how our own roles as health services researchers can be further re-imagined to facilitate partnerships through embedded research models.

Similar to Canada’s, Australia’s health system is mostly publicly funded, with responsibility for the management of hospitals devolved to our states and territories. It has a health research context that encourages, or makes mandatory in some cases, partnerships between university-based researchers and health leaders. For example, the National Health and Medical Research Council, the primary funder of medical research in Australia, runs a grant scheme for “Partnership Projects,” which involves university-based researchers teaming with organisations such as hospitals, health services, Medical Colleges, government agencies or non-government organisations on explicitly translational research projects (like CIHR’s now-concluded partnership scheme in Canada that Bowen et al^1^ mentioned). System partners must be relatively committed, and willing to provide the significant contributions that the National Health and Medical Research Council then matches. Although the strength of the partnership is considered in awarding funding, once funded there are few formal mechanisms to ensure the ongoing health of the relationship, nor evaluate whether partners receive their expected return on investment.

To conduct any research within public healthcare organisations in Australia also requires institutional consent from the site and this typically entails university-based researchers naming a principal investigator who works at the hospital and will assume responsibility for the project *in-situ*. Even with the best intentions, partnering with principal investigators at site is challenging, especially in multi-site research. We often try to forge partnerships early, during the study design phase of a research project, however, by the time ethics approval is granted it is not uncommon that partners have moved on to other roles or other organisations.^4^ The bane of health system restructuring on forming long-term partnerships with health system leaders is apparently not confined to Australia, with Bowen and colleagues’ participants also acknowledging the issue of turnover.^1^

These formal inducements to collaborate are important in getting university-based researchers and health leaders to work together; however, they offer a project-specific approach that does not necessarily promote the kinds of long-term partnerships we see as having the most benefit in driving health system improvement. Challenges to collaboration persist in the mismatch between researcher interests and the needs of the healthcare organisation, different priorities regarding the outcomes of partnerships, and researchers not understanding health system context. The “preferred” model for Bowen and colleagues’^1^ interview participants to tackle these issues was an “interface approach,” which focuses on creating spaces to “force” collaboration by providing settings where researchers and health partners develop “common agendas” for research. However, a variety of other models are currently being trialled; rather than forming around a specific project, these collaborations, built on relationships and dispositions, involve embedding research and researchers within health systems, thereby challenging the separation of academic world from the world of practice.^5^ They include translational research networks (TRNs)^6^ and variations on the theme of researcher-in-residence.^7^

TRNs are an example of providing large-scale structured support to build relationships between researchers, clinicians and consumers towards a shared aim. The Cancer Institute of New South Wales, Australia, for example, fund TRNs that have a shared vision of undertaking translational research to improve outcomes for cancer patients. TRNs provide supportive infrastructure, such as Project Officers, a range of project and professional development grants, and opportunities for meaningful dialogue to set the research agenda and integrate evidence with practice. This allows TRNs to broker and facilitate collaboration with end users and contribute to building a more research positive culture.^6^ Formal funding and infrastructure gives projects and collaborations greater institutional legitimacy. This in turn increases the opportunities for local involvement of all stakeholders, as they develop a sense of ownership of the projects.

On the other hand the researcher-in-residence model out the United Kingdom, is a variant of embedded *in situ* researchers and focuses on micro-level collaboration.^7^ Here a researcher is deployed into the healthcare system, bringing an alternative perspective and a suite of different skills to the table while being implanted for a prolonged period in a healthcare organisation. Having this relationship with end users overcomes issues of access, the perception that researchers are in an “ivory tower,” and provides time and space to work through potential differences in research-practice paradigms.^8^ Partners can share expertise and leverage the potentialities of the joint work. The researcher is better able to align research with local priorities and understand the nuances of context; these factors are often otherwise barriers to institutional buy-in to research, and implementation.

Success for both models is realised in the formation of synergistic, genuine research partnerships that thereby increase the uptake of research evidence in real world settings and lead to improved healthcare systems and organisations. In our experience, this success hinges as much on key individuals (eg, the *researcher*-in-residence^7^; the key players in a TRN^6^) and interpersonal dynamics, as the space, infrastructure and resources provided for these collaborations. It is important then to think about *who* funds embedded models of research collaboration and *how*, if these models are to be more widely implemented in the future. Both universities and healthcare systems experience perennial budgetary pressures. Perhaps even more important, though, is identifying and understanding what *qualities* and *competencies* of researchers and health system leaders contribute to the success of these collaborations. To what extent can they be cultivated or developed through training? Such research is nascent everywhere,^9^ with some of the emerging findings on qualities contributing to success summarised in Box 1. Further empirical testing of what makes these partnerships successful is something researchers can offer the continually evolving aspiration of healthcare improvement.

**Box 1. Qualities of Researchers and Key Players Associated With the Success of Embedded Research Models**

1. Resilient and flexible
2. Curious about the setting and topic
3. Self-aware
4. Able to understand and empathise with others
5. Good at managing multiple, sometimes conflicting demands
6. Patient
7. Capacity to cope with uncertainty
8. Capacity to act as a change agent in partnership with others
9. Advanced research qualifications
10. Supported by both academic institution and healthcare setting

Sources: Churruca et al,^5^ Coates and Mickan,^9^ Marshall et al,^10^ Long et al.^11^

In 2011 in a piece for *The New Yorker*, Atul Gawande,^12^ a prominent US surgeon, health services researcher and systems innovator, made an astute observation: tennis players, singers, and people who need financial advice all had coaches of some sort or another. He asked: why don’t surgeons? Instead, surgeons practice solo. Although surrounded by team members, most have no one who could fulfil the role as a coach, a person who gives advice and feedback on best practice and support for how to improve behaviour—a critical friend, a relatively unbiased observer who could see ways to improve and be able to provide that extra perspective in a psychologically safe way. A researcher-in-residence can develop a protocol, conduct an *in situ* study, disseminate findings and provide advice to all who might need it, internally and externally. Likewise, TRN key players assess the skills and needs of those in the network and establish collaborative links among stakeholders. In these cases, it is not only about *what* improvements should be made but providing support for *how* to go about this. Perhaps then the greatest benefit of embedded research is in having evidence-orientated, savvy coaches for those in the system: health leaders, clinicians, other researchers and stakeholders who, like Atul Gawande, need an arm’s length perspective—and in the case of an embedded researcher, an evidence-based one—to help make improvements.

## Conclusion

Healthcare systems are the most complex of endeavours. Despite this complexity, care quality is generally high in healthcare settings, particularly in highly resourced systems. Nevertheless, there are many opportunities for evidence-based improvements through meaningful partnerships between health system researchers and leaders. In their empirical study, Bowen et al^1^ propose a need to “re-imagine” research, and we argue one strategy for doing so involves embedding research and researchers deep inside healthcare systems. Although this approach is bound to increase healthcare complexity by adding a new kind of skills-set and new relationships to engineer, there are some clear benefits of embedded research. Evidence is still emerging about what makes these kinds of partnerships successful, although some complex mix of personal and interpersonal factors appear to be important.

## Ethical issues

Not applicable.

## Competing interests

Authors declare that they have no competing interests.

## Authors’ contributions

KC had the original idea for the manuscript and drafted numerous sections. LAE, JCL, and JB drafted sections of the manuscript. All authors critically revised the manuscript and agreed to the final version.
